# CodeMapper: semiautomatic coding of case definitions. A contribution from the ADVANCE project

**DOI:** 10.1002/pds.4245

**Published:** 2017-06-28

**Authors:** Benedikt F.H. Becker, Paul Avillach, Silvana Romio, Erik M. van Mulligen, Daniel Weibel, Miriam C.J.M. Sturkenboom, Jan A. Kors, for the ADVANCE consortium

**Affiliations:** ^1^ Department of Medical Informatics Erasmus University Medical Center Rotterdam The Netherlands; ^2^ Department of Biomedical Informatics Harvard Medical School Boston MA USA; ^3^ Department of Statistics and Quantitative Methods University of Milano–Bicocca Milan Italy; ^4^ Department of Epidemiology Erasmus University Medical Center Rotterdam The Netherlands

**Keywords:** database extraction, concept identification, semantic operations, multiple medical vocabularies, UMLS

## Abstract

**Background:**

Assessment of drug and vaccine effects by combining information from different healthcare databases in the European Union requires extensive efforts in the harmonization of codes as different vocabularies are being used across countries. In this paper, we present a web application called CodeMapper, which assists in the mapping of case definitions to codes from different vocabularies, while keeping a transparent record of the complete mapping process.

**Methods:**

CodeMapper builds upon coding vocabularies contained in the Metathesaurus of the Unified Medical Language System. The mapping approach consists of three phases. First, medical concepts are automatically identified in a free‐text case definition. Second, the user revises the set of medical concepts by adding or removing concepts, or expanding them to related concepts that are more general or more specific. Finally, the selected concepts are projected to codes from the targeted coding vocabularies. We evaluated the application by comparing codes that were automatically generated from case definitions by applying CodeMapper's concept identification and successive concept expansion, with reference codes that were manually created in a previous epidemiological study.

**Results:**

Automated concept identification alone had a sensitivity of 0.246 and positive predictive value (PPV) of 0.420 for reproducing the reference codes. Three successive steps of concept expansion increased sensitivity to 0.953 and PPV to 0.616.

**Conclusions:**

Automatic concept identification in the case definition alone was insufficient to reproduce the reference codes, but CodeMapper's operations for concept expansion provide an effective, efficient, and transparent way for reproducing the reference codes.

## 1 INTRODUCTION

In order to increase the scale of pharmacoepidemiological studies, information from multiple electronic health record (EHR) databases should be combined in a distributed, collaborative fashion.^1^ However, EHR databases use different coding vocabularies to record medical information,^2^, ^3^ such as the International Classification of Diseases version 9 Clinical Modifications (ICD‐9 CM)^4^ and version 10 (ICD‐10),^5^ the International Classification of Primary Care Version 2 (ICPC‐2),^6^ Read codes version 2 (Read‐2)^7^ and Read Clinical Terms version 3 (CTv3).^8^ In multi‐database studies, the extraction of an event typically requires several steps to achieve consistency between databases. A case definition that describes the event in the study protocol is translated into an operational definition, which is then mapped for each vocabulary into a set of codes that represents the event. The code sets are combined into queries for case identification and harmonized between databases by comparison with benchmarks from the literature and by feedback from the database custodians.

The creation of code sets for each vocabulary from the textual case definitions has been largely a manual process. Given the number and complexity of the targeted vocabularies, the mapping and harmonization process can pose an important bottleneck to the rapid implementation of collaborative epidemiological studies.^9^, ^10^ Furthermore, the rationale for including or excluding individual codes is not consistently documented, which hampers the possible reuse of code sets and queries in subsequent studies.

A previous attempt to accelerate the creation of code sets from multiple vocabularies was made in the EU‐ADR project.^10^, ^11^, ^12^ Medical concepts like diseases, symptoms, laboratory procedures, or tests were automatically identified in a case definition using the MetaMap program.^13^ Code sets representing the concepts in the targeted vocabularies were then generated using the Unified Medical Language System (UMLS),^14^ a biomedical terminology system that integrates many vocabularies including coding vocabularies commonly used in EHR databases. Whereas the identification of concepts and their projection to codes was automated, the overall workflow was not integrated or recorded to facilitate the later reuse of the mapping. The approach was also applied in other European projects like GRIP (http://www.grip‐network.org), VAESCO (http://www.vaesco.net), and EMIF (http://www.emif.eu). Similar collaborative studies in the Asian and Pacific region deal with less heterogeneous medical vocabularies (Mini‐sentinel,^15^ PRISM,^16^ VSD,^17^ and AsPEN^18^). Instead of adapting the event identification algorithm to the different databases, databases can also be mapped to a standardized coding system. A single event identification algorithm can then be used in different databases. This approach has been pursued in OMOP^19^ and OHDSI.^20^

We present a web application called CodeMapper, which has been developed in the Accelerated Development of Vaccine Benefit–Risk Collaboration in Europe (ADVANCE) project (http://www.advance‐vaccines.eu). It is based on the EU‐ADR approach and assists in mapping case definitions to code sets from different vocabularies while keeping a record of the complete mapping process. We evaluate the application by comparing code sets that were automatically generated by CodeMapper with reference code sets that were manually created in a previous epidemiological study.

**KEY POINTS**

- Web application to assist mapping case definitions to code sets
- Effective and efficient manual revision operations on medical concepts
- Automatic identification of medical concepts in case definition
- Automatic creation of code sets from multiple medical terminologies

## 2 METHODS

**Figure 1:**
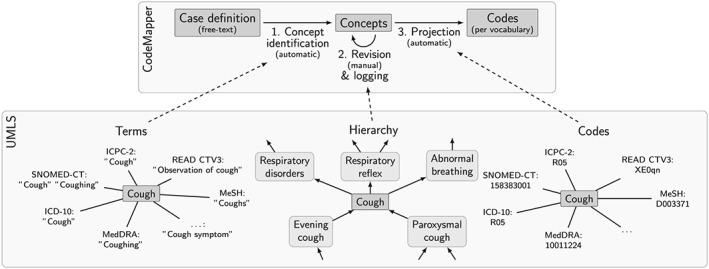
Key phases of CodeMapper (top) and the usage of information from the UMLS Metathesaurus, exemplified by the concept for *Cough* with CUI C0010200 (bottom). Terms from the Metathesaurus drive the automatic identification of concepts in the free‐text case definition. Hierarchical information about concepts in the Metathesaurus is used to retrieve related concepts during revision of the mapping. Information in the Metathesaurus is used to project the selected concepts to codes from the targeted vocabularies.

CodeMapper's mapping approach consists of 3 phases (Figure 1, top). First, medical concepts are automatically identified in a free‐text case definition. The user can then revise the set of medical concepts by adding or removing concepts or by expanding a concept to more general or more specific concepts. For example, the concept *Coughing* can be expanded to more general concepts such as *Respiratory disorders* and *Abnormal breathing*. Expanding it to concepts that are more specific results in subtypes of coughing such as *Paroxysmal cough* and *Evening cough*. Finally, each concept is represented by (possibly several) codes in the targeted vocabularies, and the projection of the concepts to codes forms the result of the mapping process. In this section, we will describe the mapping approach, the CodeMapper application, and an evaluation of the approach.

### 2.1 Mapping approach

CodeMapper builds upon information from the Metathesaurus of the UMLS. The Metathesaurus is a compendium of many medical vocabularies, which have been integrated by assigning equivalent codes and terms from different source vocabularies to the same concepts. Each concept in the UMLS is identified by a concept unique identifier (CUI). For example, the concept *Coughing* (CUI: C0010200) is among others associated with the codes 786.2 (ICD‐9 CM), R05 (ICD‐10), and XC07I (CTv3). The Metathesaurus contains more than 1 million concepts connected to codes from 201 vocabularies. Each concept is assigned to 1 or more of 127 semantic types, which define broad conceptual categories like *Disease or syndrome*, *Finding*, or *Substance*. To provide even broader structure, semantic types are combined into 15 semantic groups.^21^ We used version 2016AA of the UMLS in this evaluation.

The automatic concept identification of CodeMapper is based on lexical information from the Metathesaurus. The lexical information of a concept consists of terms that can be used in free‐text to refer to that concept (Figure 1, bottom left). We compiled a dictionary for the concepts in the semantic groups *Anatomy*, *Chemicals & Drugs*, *Disorders*, *Genes & Molecular Sequences*, *Living Beings*, *Phenomena*, *Physiology*, and *Procedures* of nonsuppressible, English terms from the following vocabularies: Medical Subject Headings (MeSH),^22^ Medical Dictionary for Regulatory Activities (MedDRA),^23^ Systematized Nomenclature of Medicine—Clinical Terms (SNOMED‐CT),^24^ ICD‐9 CM, ICD‐10 CM, ICPC‐2, and CTv3. Our text‐indexing engine Peregrine uses this dictionary to identify medical concepts in the case definition.^25^

CodeMapper provides two operations to improve the sensitivity of the mapping by expanding a concept to more general or more specific concepts, based on the hierarchical relationships in the Metathesaurus. Hierarchical relationships connect concepts that are more general or more specific in meaning (Figure 1, bottom center). For example, the concept for *Coughing* is connected to the more general concept *Respiratory Disorders*, and to the more specific concept *Paroxysmal cough*. To expand a concept in CodeMapper, all concepts that have a more general or more specific relationship with it are identified and displayed in the application for selection by the user. Hierarchical relationships in the Metathesaurus are inherited from the source vocabularies or defined in the Metathesaurus.^26^ Both types of hierarchical relationships are taken into account for concept expansion.

The projection of concepts to code sets from the targeted vocabularies follows the identification of equivalent codes in the Metathesaurus (Figure 1, bottom right).

### 2.2 Application

The CodeMapper application is implemented as a web application and freely available for noncommercial use (https://euadr.erasmusmc.nl/CodeMapper). CodeMapper has three screens. On the first screen, the user enters a clinical case definition of an event as free text. Medical concepts are automatically identified in the text and highlighted inline. By default, only concepts that belong to the semantic group of *Disorders* are preselected for further processing in the application, but the user can select and deselect any identified concept depending on their relevance for the described event.

**Figure 2:**
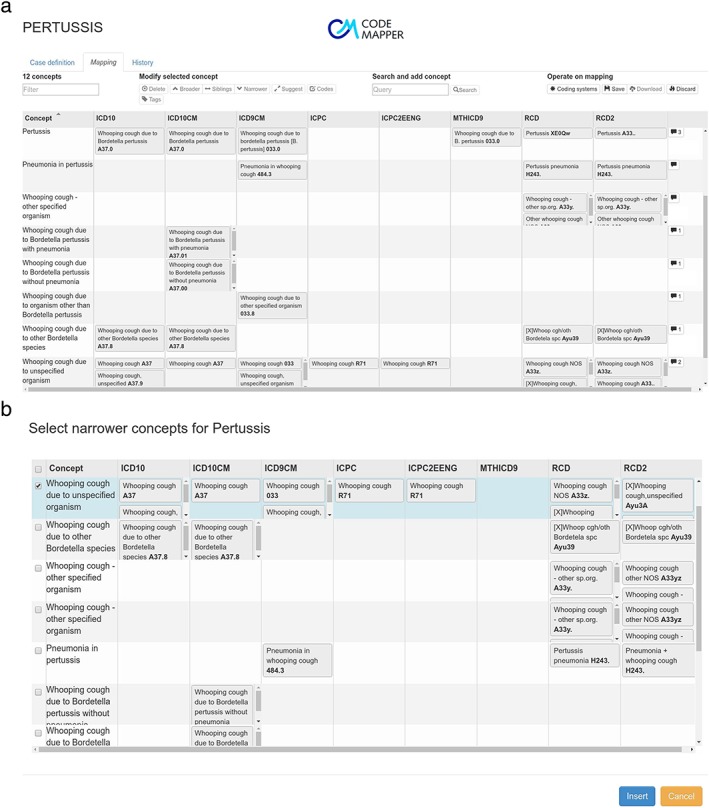
(a) The second screen of the CodeMapper application provides operations to revise the concepts of a mapping. The mapping is displayed as a table. The cells show the code names from the vocabulary stated in the column that correspond to the concept of the row. Individual codes are shown when hovering the terms. The balloons in the last column indicate the number of comments attached to a concept. (b) Example of the operation for concept expansion: A list of concepts that are related to pertussis as more specific is displayed for the selection and insertion in the mapping. [Color figure can be viewed at wileyonlinelibrary.com]

The second screen displays the mapping as a table with one row for each medical concept, and one column for each targeted vocabulary (Figure 2). Each cell contains the names of the codes that are used to represent the medical concept of the row in the targeted vocabulary of the column. The codes are displayed when the names are hovered over with the mouse. Several user operations are available for revising the mapping. The user can remove concepts from the mapping, search and add concepts, or retrieve more general and more specific concepts. The retrieved concepts are shown in a list and can be selected by the user for inclusion in the mapping. The user can also add or remove vocabularies that should be targeted by the mapping. After every operation, the codes are automatically updated and displayed in the table.

The third screen shows a list of all operations that have been performed, for later traceability of the mapping process. When the user saves the mapping, he or she is asked to provide a summary of the modifications, which is incorporated into the mapping history. After saving, the mapping and history lists are available to other users of the application. Comments can be attached to concepts to capture the discussion about the mapping. Concepts can be categorized by tags. Finally, the user can download the mapping as a spreadsheet file, for example, to incorporate the codes into extraction queries. The spreadsheet file comprises the original free‐text case definition, the concepts of the mapping, the codes for the targeted vocabulary, and the full history of the mapping process.

### 2.3 Evaluation

We evaluated the effectiveness of CodeMapper's approach for creating realistic code sets, by comparing code sets that were generated with CodeMapper with manually created reference code sets. We used the case definitions and reference code sets from the FP‐7 funded SAFEGUARD project (http://www.safeguard‐diabetes.org),^27^ which was conducted in nine different EHR databases in the EU and US. The full study protocol, which includes the case definitions, can be found in the EU‐PAS registry (http://www.encepp.eu/encepp/viewResource.htm?id=8323). This project was selected for the variety of mapped events and the range of targeted vocabularies. The manual mapping process consisted of deriving operational definition from the textual case definition, choosing codes from the targeted vocabularies without the use of the Metathesaurus, and refining the code set based on feedback from database custodians. The reference mappings also contained exclusion codes, which were not considered in the evaluation because they were not generally derived from the case definitions.

SAFEGUARD studied nine events: acute pancreatitis, bladder cancer, hemorrhagic stroke, heart failure, ischemic stroke, acute myocardial infarction, pancreatic cancer, sudden cardiac death, and ventricular arrhythmia. One event (sudden cardiac death) was excluded from the evaluation because of several missing code sets, and another (heart failure) because the case definition contained only a short symptomatic description of the event, unrelated to the codes representing the event. The events were mapped for nine EHR databases with four vocabularies: Medicare, PHARMO, HSD, and regional EHR databases from Lombardy and Puglia (all these databases use ICD‐9 CM), GePaRD (ICD‐10, German modifications), IPCI and BIFAP (both ICPC‐2 and keywords), and CPRD (Read‐2). We selected the code sets for Medicare for ICD‐9 CM as the reference because it contained less database‐specific additions than the other code sets using ICD‐9 CM. The codes for GePaRD are contained by the ICD‐10 and ICD‐10 CM vocabularies in the UMLS, so we combined the codes generated by CodeMapper for these vocabularies. The Metathesaurus covers only CTv3 and not Read‐2. To generate codes for Read‐2, a translation table between Read‐2 and CTv3 was integrated into CodeMapper (provided by the Health and Social Care Information Centre). Codes from the IPCI mapping were trimmed to 3 digits to adjust for the database‐specific codes in IPCI.

Overall, the reference code sets contained 420 codes (Table 1). The size of the reference code sets varied widely between vocabularies: on average, the code sets for Read‐2 contained 48.3 codes, whereas the code sets for ICPC‐2 contained 1.1 codes. This discrepancy is firstly due to the differences of granularity of the vocabularies (Read‐2 has 77290 codes in the Metathesaurus, ICPC‐2 only 1397). Second, the queries to the IPCI database (to which the ICPC‐2 code sets are targeted) were supported by keyword searches on the free‐text portion of the IPCI medical records and additional exclusion criteria.

**Table 1:** Number of words in the case definitions and number of codes in the reference set. The numbers of exclusion codes are given in brackets

	Case definition (word count)	Codes			
Event		ICD‐9	ICD‐10	ICPC‐2^a^	Read‐2
Acute pancreatitis	49	1 (0)	6 (0)	1 (0)	7 (0)
Bladder cancer	87	12 (0)	12 (0)	1 (3)	91 (0)
Hemorrhagic stroke	48	3 (2)	22 (2)	1 (2)	36 (0)
Ischemic stroke	53	10 (0)	11 (0)	2 (1)	20 (0)
Acute myocardial infarction	39	11 (1)	7 (0)	1 (6)	–^b^
Pancreatic cancer	19	8 (0)	9 (0)	1 (1)	109 (0)
Ventricular arrhythmia	234	5 (0)	5 (0)	1 (1)	27 (0)
Sum	529	50 (3)	72 (2)	8 (14)	290 (0)
Average	75.57	7.14 (0.43)	10.29 (0.29)	1.14 (2.0)	48.33 (0.0)

Additional text‐based queries for IPCI database.

Text‐based query only for GePaRD database.

**Figure 3:**
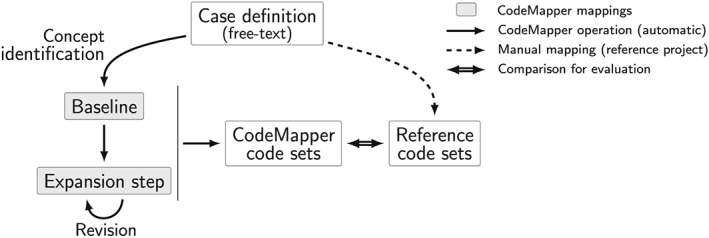
Automatic evaluation of CodeMapper. Reference code sets were created manually for each targeted vocabulary from the free‐text case definition of an event. The baseline mappings and expansion steps were generated automatically from the same case definition using the operations available in CodeMapper.

Different code sets were generated fully automatically by CodeMapper for the events of the reference project based on the same case definitions. The baseline code sets resulted from the concepts identified in the case definition (Figure 3). We then simulated the actions of an “informed user” who seeks to improve the sensitivity of the mapping. We assumed that this user would expand the concepts and, from all possible concepts that are more general or more specific, would only retain those that are relevant to the event. Based on the reference set we were able to automatically simulate the “informed user's” actions. The resultant set of concepts defined a new code set, which always contained all codes from the preceding code set. We simulated four of these expansion steps on successive concept sets.

For each target vocabulary and event, the generated code set was compared with the reference code set. We determined the number of true‐positive codes (TP), false‐positive codes (FP), and false‐negative codes (FN), and computed sensitivity (TP / (TP + FN)) and positive predictive value (PPV) (TP / (TP + FP)). We report for each vocabulary the sensitivity and PPV averaged over all events in the reference set.

### 2.4 Error analysis

**Figure 4:**
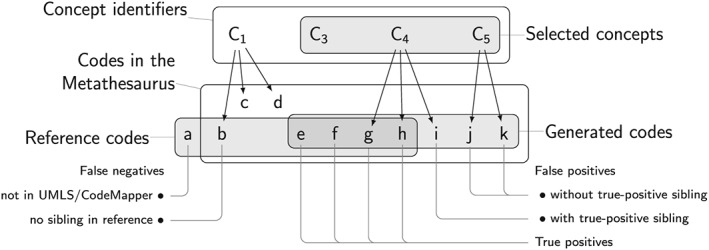
Categories of false negatives and false positives in the error analysis. Two codes are siblings if they are associated with the same concept.

We then carried out an automatic error analysis of the false‐positive and false‐negative codes after the third expansion step (Figure 4). The definitions of the error categories were based on the notion of sibling codes*:* 2 codes are siblings if they are linked to the same concept in the Metathesaurus. For false negatives, we distinguished between codes that are not contained in the Metathesaurus and codes whose siblings are not in the reference sets. False‐positive codes were categorized as having or not having a true‐positive sibling code.

## 3 RESULTS

### 3.1 Baseline

The baseline mapping created by CodeMapper had an average sensitivity of 0.246 for reproducing the reference code sets (Table 2).The average PPV of the baseline mapping was 0.420. Without filtering by the semantic group of *Disorders*, the number of concepts would increase from 46 to 77 without affecting the sensitivity of the generated code sets.

**Table 2:** Number of concepts and performance measures of the mappings in the evaluation. Numbers per vocabularies are macro‐averages over all events

Revision (number of concepts)		ICD‐9	ICD‐10	ICPC‐2	Read‐2	Average
Baseline (46)	Sensitivity	0.300	0.195	0.357	0.131	0.246
	PPV	0.387	0.380	0.500	0.411	0.420
Expansion step 1 (183)	Sensitivity	0.858	0.848	1.000	0.568	0.818
	PPV	0.483	0.558	0.762	0.729	0.633
Expansion step 2 (297)	Sensitivity	0.914	1.000	1.000	0.846	0.940
	PPV	0.463	0.509	0.762	0.749	0.621
Expansion step 3 (335)	Sensitivity	0.929	1.000	1.000	0.882	0.953
	PPV	0.462	0.498	0.762	0.742	0.616

### 3.2 Concept expansion

The sensitivity of the baseline mapping greatly improved in the first expansion step, to 0.818. Sensitivity further increased in the second (0.940) and third (0.953) expansion steps. All ICPC‐2 codes were produced after the first expansion step and all ICD‐10 codes were produced after the second step. The sensitivity increased incrementally for Read‐2 and ICD‐9 CM. The PPV improved after one expansion step (0.633) and decreased slightly after 2 (0.621) and 3 (0.616) expansion steps. The performance did not improve further in a fourth expansion step. The sensitivity was lower after three expansion steps when using only hierarchical relationships that were inherited from the source vocabularies (0.928) or defined in the Metathesaurus (0.879).

### 3.3 Error analysis

False‐positive codes were generated in all vocabularies after the third expansion step (Table 3). Most false‐positive codes had true‐positive siblings (*N* = 164; 70.1%). False‐positive codes without true‐positive siblings (*N* = 70; 29.9%) resulted from the initial concept identification step because the concept expansion steps (simulating the informed user) added only concepts with true‐positive codes.

**Table 3:** Number of false‐positive codes after three expansion steps by vocabulary and error category, and their percentage of all false‐positive codes

Vocabulary	FP category	Count	Percentage
ICD‐9 CM	With TP sibling	52	22.2
	No TP sibling	22	9.4
ICD‐10	With TP sibling	66	28.2
	No TP sibling	30	12.8
ICPC‐2	With TP sibling	3	1.3
	No TP sibling	1	0.4
READ‐2	With TP sibling	43	18.4
	No TP sibling	17	7.3
Overall	With TP sibling	164	70.1
	No TP sibling	70	29.9

False‐negative codes occurred only for Read‐2 and ICD‐9 CM (Table 4). Most false‐negative codes did not have any siblings in the reference set (*N* = 24; 68.6%), suggesting that the code was added to the reference set due to database specific needs. Other false‐negative Read‐2 codes were not contained in the conversion table from CTv3 to Read‐2 codes, or the CTv3 codes corresponding with the Read‐2 codes were not in the Metathesaurus (*N* = 11; 31.4%).

**Table 4:** Number of false‐negative codes after three expansion steps by vocabulary and error category, and their percentage of all false‐negative codes

Vocabulary	FN category	Count	Percentage
READ‐2	No sibling in reference	19	54.3
	Not in UMLS	11	31.4
ICD‐9 CM	No sibling in reference	5	14.3
Overall	No sibling in reference	24	68.6
	Not in UMLS	11	31.4

A mapping constructed to maximize sensitivity by selecting concepts to generate all available codes from the reference sets had a sensitivity of 0.991 and PPV of 0.733.

## 4 DISCUSSION

In this article, we presented the CodeMapper web application that assists in the mapping of textual case definitions to code sets from multiple vocabularies, which is often a bottleneck in the implementation of epidemiological multi‐database studies. We showed the effectiveness of CodeMapper's approach by simulating an informed usage of the application.

Creating a mapping only by the automatic identification of medical concepts in the case definition was insufficient for reproducing the reference code sets (sensitivity 0.246). The mapping process cannot be replaced by a simple indexing step. However, the goal of CodeMapper is to support an informed user in creating such mappings, and CodeMapper's operations for concept expansion provide an effective and efficient way for this. The reference code sets were regenerated with a sensitivity of 0.953 and PPV of 0.616 after only three expansion steps. The reference codes for ICPC‐2 were even completely regenerated after the first expansion step and the reference codes for ICD‐10 after the second expansion step. The sensitivity was increasing between expansion steps because codes were always retained in subsequent steps. The sensitivity was lower for more granular vocabularies, where more expansion steps were required. The increase of sensitivity came at the costs of a slight decrease in PPV, which is a consequence of the introduction of false‐positive codes that are siblings of newly added true‐positive codes.

The performance of the mapping that simulates maximal sensitivity (0.991 with associated PPV of 0.733) forms an upper bound of CodeMapper's performance in regenerating the reference code sets. The imperfect sensitivity is due to reference codes that are missing in the UMLS or in the mapping between Read‐2 and CTv3. The moderate PPV may be due to inconsistencies in the reference code sets or the Metathesaurus. The reference code sets may be inconsistent between vocabularies for two reasons. First, the inclusion of one code in the reference mapping did not always imply the inclusion of all sibling codes in the targeted vocabularies, which is reflected by the large number of false positives with true‐positive siblings. Second, different code sets were created for databases with the same vocabularies, which can be necessary to compensate for characteristics of the databases. For example, when an event is only available as inpatient diagnosis in one database, a drug that is usually prescribed in case of the event in outpatient setting can be included in the query as a proxy*.* Such database‐specific additions also explain some false‐negative codes without siblings in the reference set. Inconsistencies in the Metathesaurus such as missing identification of equivalent codes and incomplete coverage of vocabularies have been discussed before.^28^, ^29^, ^30^, ^31^

The SAFEGUARD reference set contained only codes for diagnoses but no codes for laboratory, imaging or ECG results. CodeMapper's operations for concept expansion would not be suitable for generating codes for diagnostic tests because the corresponding concepts are not hierarchically related to the concepts for the diagnoses given in the case definition. However, these result codes can be generated using CodeMapper's approach if the concepts for diagnostic tests are mentioned in the case definition.

When exclusion criteria are indicated in the case definition, CodeMapper's approach can be applied to map them to codes, but they must then manually be marked for exclusion to inform the data extraction process. Automatic negation extraction^32^ could be used to automate the identification of exclusion criteria in the case definition.

The use of the Metathesaurus in CodeMapper's approach brings practical limitations with it. Differences in granularity between vocabularies can affect the consistency of the generated code sets. This problem could be identified by incorporating information about code usage in EHR databases into CodeMapper. Vocabularies that are not part of the UMLS can only be targeted with CodeMapper by integrating additional mapping tables. Moreover, database‐specific code sets cannot be maintained easily because code sets are generated per vocabulary.

CodeMapper has been applied to the mapping of 45 events in the ADVANCE project so far. The automatic concept identification and revision operations allowed a quick drafting and interactive exploration of the code sets, without requiring extensive knowledge of each targeted vocabulary. Feedback from medical experts and database custodians, and harmonization between databases were crucial to identify missing codes and concepts. Feedback was collected in CodeMapper as comments to inform the subsequent revision of the mappings. Together with the detailed history of all steps that resulted in the mapping, CodeMapper facilitated an integrated and transparent management of the overall mapping process.

In conclusion, the CodeMapper web application constitutes a single entry point for the different phases of the terminology mapping process for multi‐database studies. The expansion operations provide a more efficient and systematic way to add relevant related codes to the mapping than browsing the source vocabularies. The integration of the mapping process into a single application and the recording of user operations make the mapping process traceable and the mappings more suitable for reuse in subsequent studies.

## ETHICS STATEMENT

The authors state that no ethical approval was needed.

## CONFLICT OF INTEREST

The authors declare that they have no conflict of interest.
